# Error in Table 2

**DOI:** 10.1001/jamanetworkopen.2019.8448

**Published:** 2019-07-10

In the Original Investigation titled “Effectiveness of Targeted Insulin-Adherence Interventions for Glycemic Control Using Predictive Analytics Among Patients With Type 2 Diabetes: A Randomized Clinical Trial,”^1^ published March 15, 2019, in Table 2, the data in the last 2 columns of the bottom row are relative change, not relative risk. This article has been corrected.^1^

## References

[1] Lauffenburger JC, Lewey J, Jan S, Effectiveness of targeted insulin-adherence interventions for glycemic control using predictive analytics among patients with type 2 diabetes: a randomized clinical trial. JAMA Netw Open. 2019;2(3):. doi:10.1001/jamanetworkopen.2019.0657 PMC6484630 30874782

